# *Mycobacterium tuberculosis* KasA as a drug target: Structure-based inhibitor design

**DOI:** 10.3389/fcimb.2022.1008213

**Published:** 2022-09-15

**Authors:** Reshma S. Rudraraju, Samer S. Daher, Ricardo Gallardo-Macias, Xin Wang, Matthew B. Neiditch, Joel S. Freundlich

**Affiliations:** ^1^ Department of Microbiology, Biochemistry and Molecular Genetics, New Jersey Medical School, Rutgers University, Newark, NJ, United States; ^2^ Department of Pharmacology, Physiology, and Neuroscience, New Jersey Medical School, Rutgers University, Newark, NJ, United States; ^3^ Department of Immunology and Infectious Diseases, Harvard University T.H. Chan School of Public Health, Boston, MA, United States; ^4^ Department of Medicine, Center for Emerging and Re-emerging Pathogens, New Jersey Medical School, Rutgers University, Newark, NJ, United States

**Keywords:** *mycobacterium tuberculosis*, KasA, β-ketoacyl synthase, structure-based drug discovery, medicinal chemistry

## Abstract

Recent studies have reported the β-ketoacyl-acyl carrier protein KasA as a druggable target for *Mycobacterium tuberculosis*. This review summarizes the current status of major classes of KasA inhibitors with an emphasis on significant contributions from structure-based design methods leveraging X-ray crystal structures of KasA alone and in complex with inhibitors. The issues addressed within each inhibitor class are discussed while detailing the characterized interactions with KasA and structure-activity relationships. A critical analysis of these findings should lay the foundation for new KasA inhibitors to study the basic biology of *M. tuberculosis* and to form the basis of new antitubercular molecules of clinical significance with activity against drug-sensitive and drug-resistant infections.

The cell wall of *Mycobacterium tuberculosis* plays a defining role in terms of its interactions with the host immune system and with antitubercular drugs (Rahlwes et al., 2019; Dulberger et al., 2020). Its cell wall is comprised of peptidoglycan (PG) covalently attached to the heteropolysaccharide arabinogalactan (AG) *via* phosphoryl-*N*-acetylglucosaminosyl-rhamnosyl linkage units (P-GlcNAc-Rha). AG is in turn esterified at its non-reducing ends to long α-alkyl β-hydroxy fatty acids known as mycolic acids (MAs). This PG-AG-MA triumvirate is viewed as the inner leaflet of a mycomembrane with the outer leaflet primarily comprised of non-covalently bound mycolic acids, trehalose monomycolate, and trehalose dimycolate (Zuber et al., 2008; Hoffmann et al., 2008; Marrakchi et al., 2014). MAs are synthesized by the fatty acid synthase-I (FAS-I) and fatty acid synthase-II (FAS-II) proteins (**Figure 1**) (Brennan and Nikaido, 1995; Cole et al., 1998). The FAS-I cycle generates fatty acids (FAs) with shorter chain fragments ranging up to C_16_-C_18_ and C_24_-C_26_ (Takayama et al., 2005), in which the latter corresponds to the α-branch found in MAs. The FAS-II cycle further elongates the FAs to afford C_50_-C_56_ meromycolates (Marrakchi et al., 2014). Pks13 condenses the FAS-I cycle fatty acid product and the FAS-II cycle meromycolic acid product to form the MA (Portevin et al., 2004; Leger et al., 2009; Gavalda et al., 2009). Despite the fact that the two cycles differ in the carrier protein, molecular organization, and substrates, they perform analogous reaction sequences with an iterative series of steps relying on consecutive additions of a two-carbon (acetate) unit ultimately from malonyl-Coenzyme A (CoA) to an acyl moiety. The malonyl group is transferred from malonyl-CoA to the mycobacterial acyl carrier protein (AcpM) by malonyl CoA-ACP transacylase (MtFabD, Rv2243) to form malonyl-AcpM. The condensation of malonyl-AcpM with acyl-CoA is catalyzed by the β-ketoacyl-ACP synthase III (MtFabH, Rv0533c) to form β-ketoacyl-AcpM and, thus, link the FAS-I and FAS-II cycles. Generally, there are four main enzymes involved in each cycle of elongation. The nicotinamide adenine dinucleotide phosphate-dependent 3-ketoacyl-acyl carrier protein reductase (MabA, Rv1483) reduces the β-keto group. Heterodimeric (3R)-hydroxyacyl-ACP dehydratase (HadAB, Rv0635 – Rv0636, and HadBC, Rv0636 – Rv0637) subsequently dehydrates the resulting β-hydroxy intermediate into enoyl-AcpM. Then, the nicotinamide adenine dinucleotide hydrogen (NADH)-dependent *trans*-2-enoyl-ACP reductase (InhA, Rv1484) reduces enoyl-AcpM into acyl-AcpM. Subsequent cycles of elongation are carried out by KasA (Rv2245) and KasB (Rv2246) (**Figure 2**) that elongate the acyl-AcpM by two carbons to form the β-ketoacyl-AcpM. KasA and KasB are 67% identical and 86% similar by protein sequence, and they exhibit differential substrate preferences (Bhatt et al., 2007; Bhatt et al., 2007).

**Figure 1:**
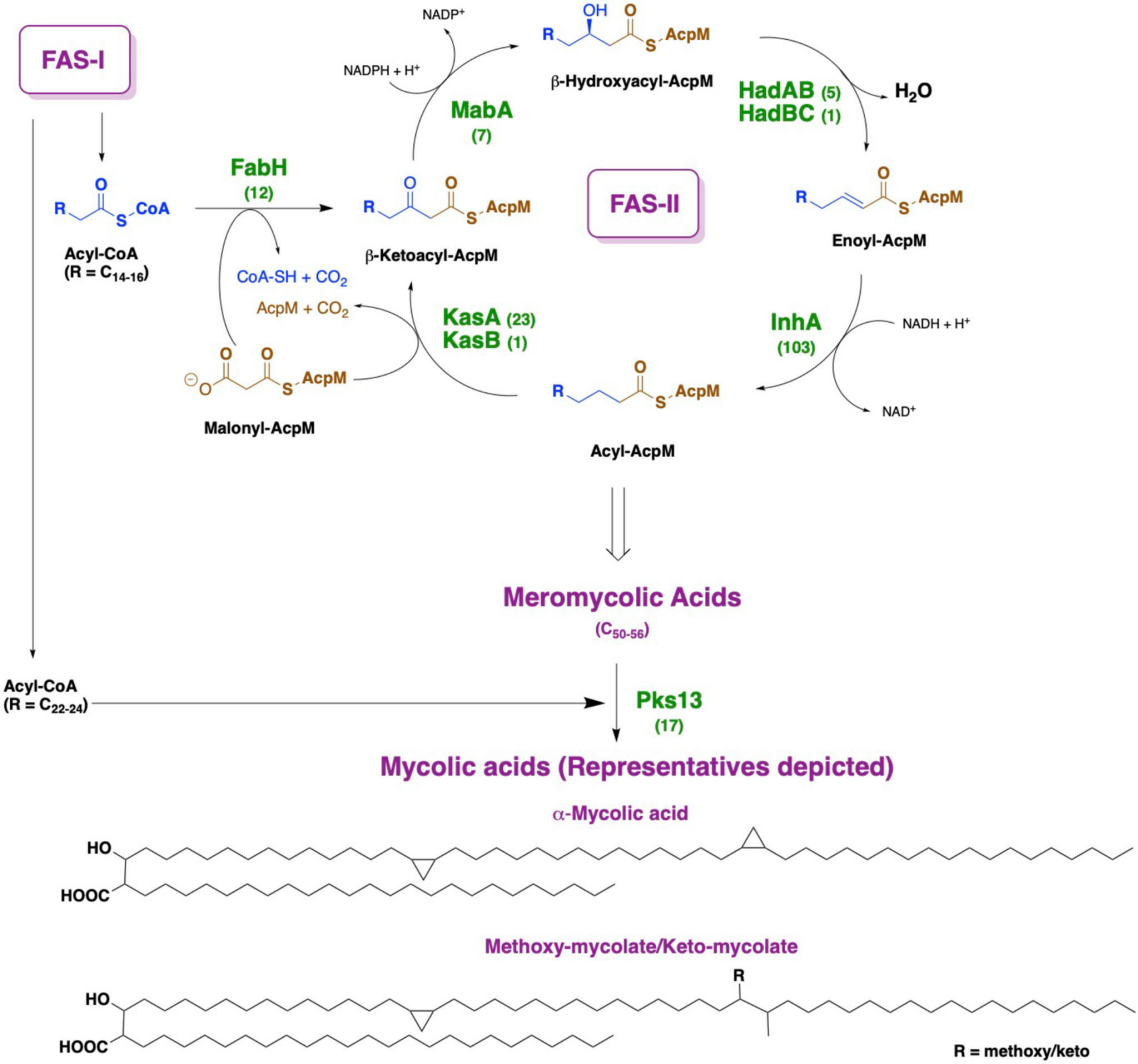
Depiction of mycolic acid biosynthesis through the FAS-I and FAS-II pathways. AcpM: mycobacterial acyl carrier protein. By each protein name, the number of corresponding X-ray crystal structures deposited in the Protein Data Bank is annotated.

**Figure 2:**
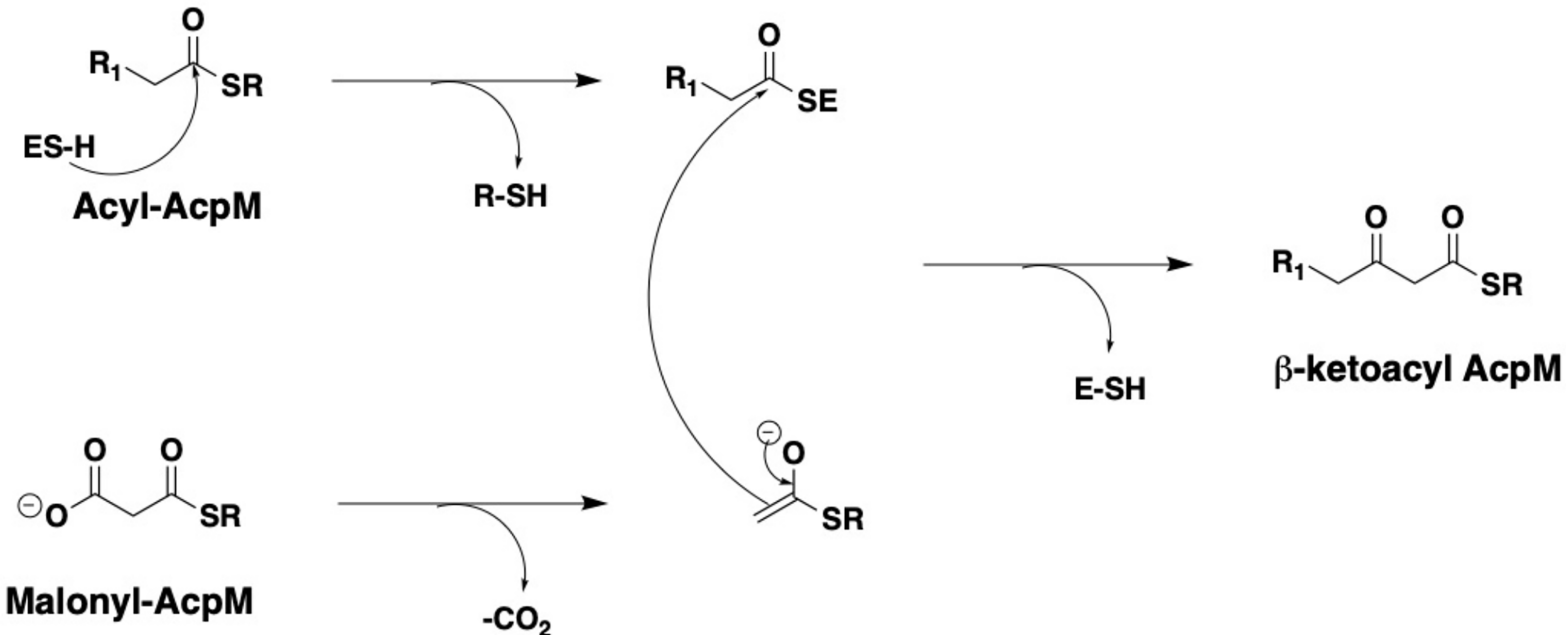
General catalytic mechanism of the KasA and KasB enzymes.

MA synthesis inhibition has represented a fertile ground for antitubercular drug targeting (Abrahams and Besra, 2020; Fernandes et al., 2022; Dartois and Rubin, 2022), and this is undoubtedly due to the availability of X-ray crystal structures of many involved proteins (**Figure 1**) in the Protein Data Bank (www.rcsb.org). This strategy is highly favorable because developed inhibitors are expected to lack mechanism-based toxicity as mammals rely primarily on a FAS-I system (Bhatt et al., 2007). For instance, isoxyl and thioacetazone are known to target the HadAB/HadBC (3R)-hydroxyacyl-ACP dehydratase complex, whereas isoniazid and ethionamide prevent the reduction of enoyl-AcpM by targeting InhA (Banerjee et al., 1994; Vilcheze et al., 2006). We highlight the fact that an *M. tuberculosis* β-ketoacyl synthase is not currently the target of a tuberculosis drug. Thus, a therapeutic inhibiting KasA should be clinically useful versus both drug-sensitive and drug-resistant infections.

## KasA is an essential and vulnerable drug target *in vitro*

*kasA* has been identified as an essential gene for the *M. tuberculosis* laboratory strain H37Rv under *in vitro* growth conditions *via* multiple transposon-based approaches, including the original transposon site hybridization method (TRaSH) (Sassetti et al., 2003), deep sequencing of transposon insertions (TnSeq) (Griffin et al., 2011; Zhang et al., 2012), and a more comprehensive TnSeq analysis (DeJesus et al., 2017) to account for TA insertability with a hidden Markov model. In contrast, the *in vitro* essentiality of *kasB* appears to be dependent on the experimental conditions (growth media and nature of genetic disruption) (Minato et al., 2019). Extensive studies show no evidence of *kasA* as a conditional non-essential gene, indicating that the *in vitro* requirement for *kasA* is independent of culture conditions. Consistent with these findings with laboratory strains, *kasA* was also revealed as essential for *in vitro* cultures of clinical isolates belonging to the most prevalent lineages: Euro-American, East Asian, and Indo-Oceanic (Carey et al., 2018). A gene vulnerability study based on the mycobacterial CRISPRi system (Rock et al., 2017) identified *kasA* as a vulnerable gene in both laboratory strain H37Rv and clinical isolate HN878 (Bosch et al., 2021). These studies indicate that *M. tuberculosis in vitro* growth is highly dependent on *kasA* and the FAS-II pathway in general. The data supporting the *in vitro* essentiality and vulnerability of *kasA* in *M. tuberculosis* have created a significant impetus to attain *in vivo* validation of this drug target.

## Protein crystallography has provided key insights into KasA catalysis

**Figure 3:**
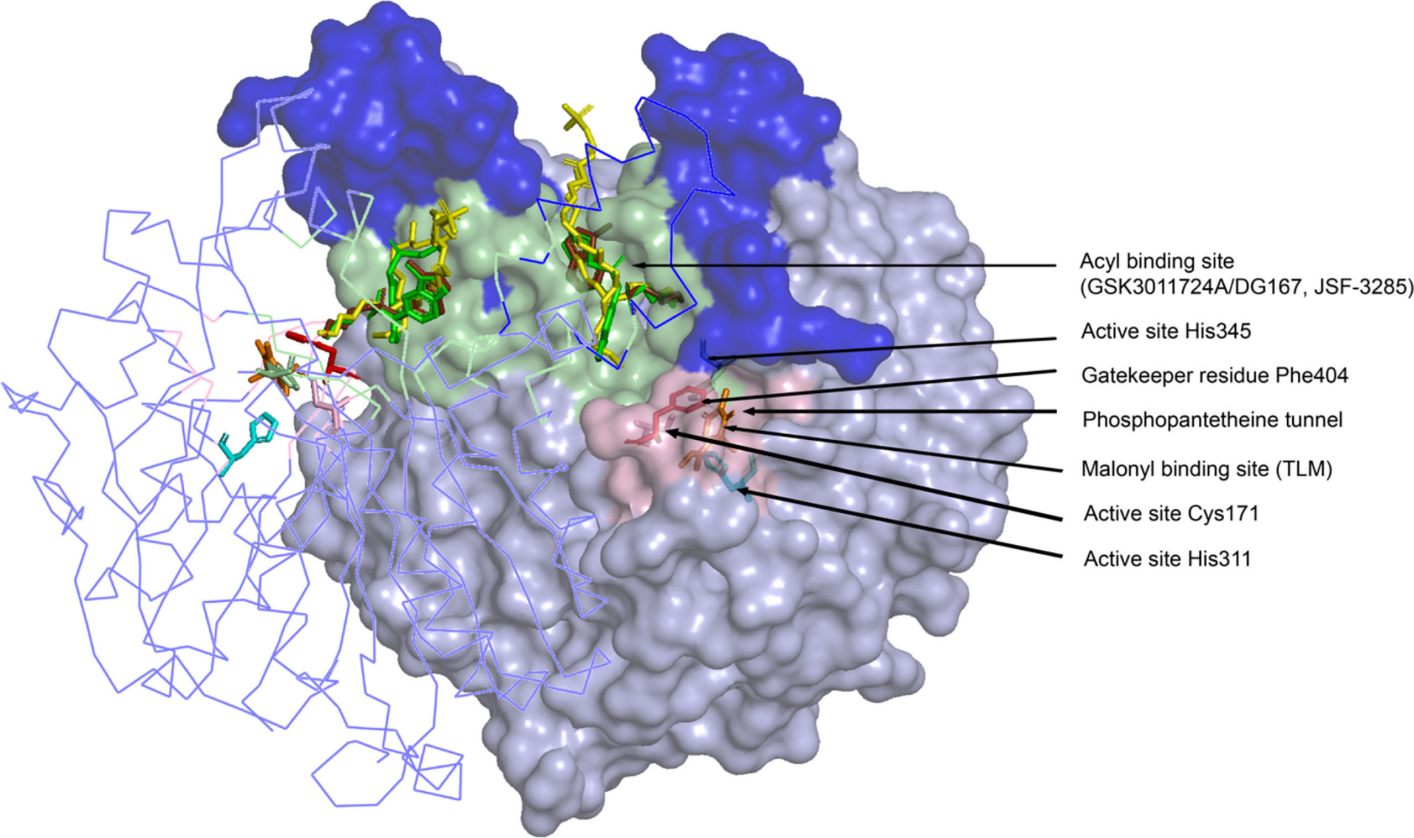
Crystal structure of the KasA dimer  with inhibitors bound (GSK3011724A/DG167, JSF-3285, TLM) at different binding sites. The monomer on the left is represented as a ribbon tracing the alpha carbons and the monomer on the right is depicted as a surface. The dark blue surface indicates the cap region of KasA excluding the acyl channel whereas the light blue surface indicates the core domain; Light green surface, acyl binding site; pink surface, malonyl binding site; yellow sticks; phospholipid bound to KasA (PDB ID 4C6W); green sticks; DG167 (PDB ID 5W2P); brown sticks; JSF-3285 (PDB ID 6P9L); orange sticks; TLM (PDB ID 4C6U); pink sticks, Cys171; red sticks, Phe404; cyan sticks, His 311 and His 345. For clarity, only the KasA dimer from PDB ID 6P9L is shown.

**Figure 4:**
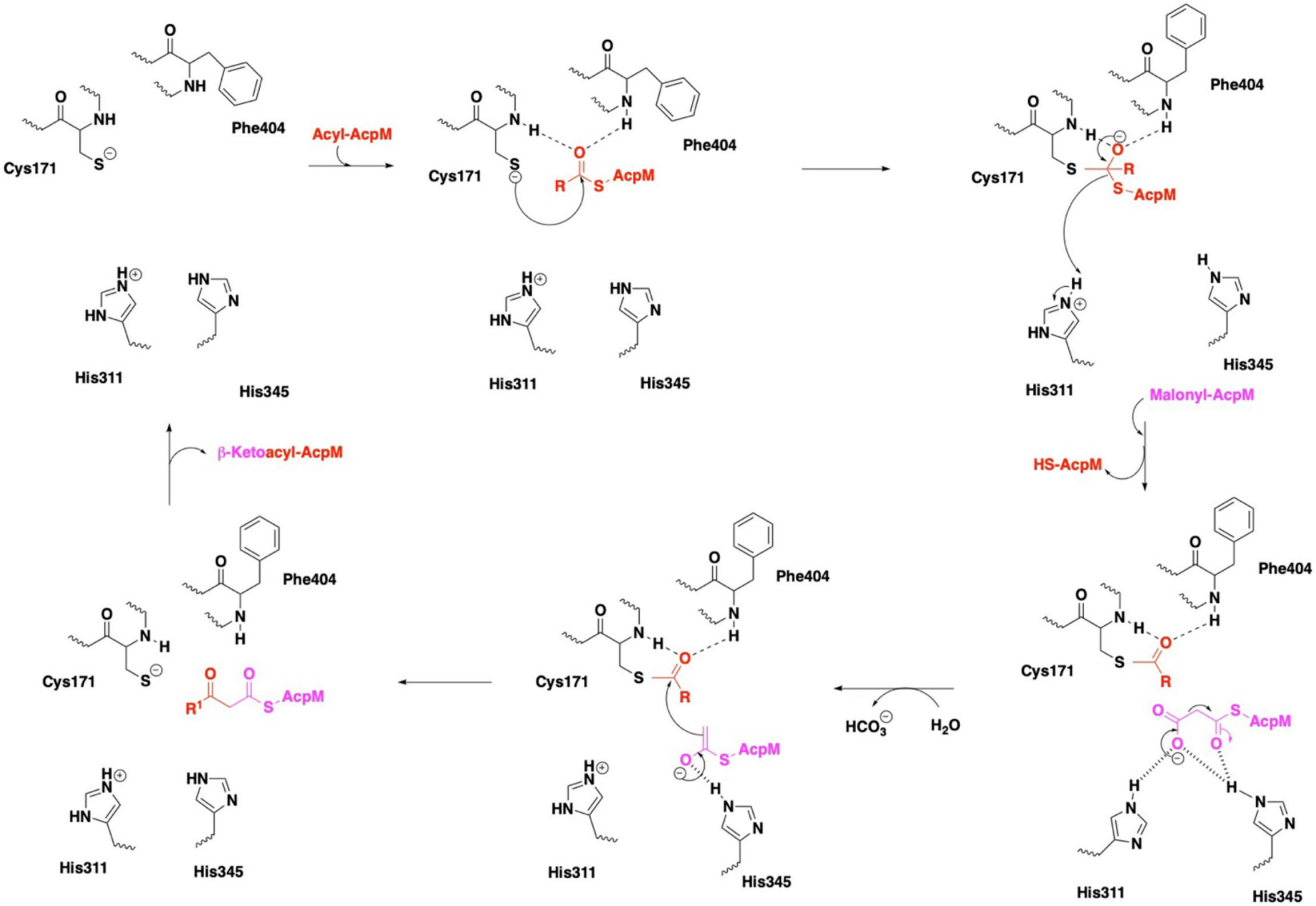
Mechanism of KasA catalysis with emphasis on the catalytic triad. In the KasA resting state, Cys171 and His311 are proposed to be deprotonated and protonated, respectively. Acylation of KasA occurs by nucleophilic attack of Cys171 on the acyl-AcpM. Several theories have been offered to explain the decarboxylation step, and it is unclear as to the protonation state of His311. The ensuing condensation of this enolate with the Cys171 bound acyl moiety occurs to elongate the acyl chain length by two carbons and then release the product.

X-ray crystallographic analyses of KasA alone (or apo) (Luckner et al., 2009; Kumar et al., 2018), KasA-C171Q in complex with fortuitously-bound phospholipid (Luckner et al., 2009; Schiebel et al., 2013), and KasA bound to small molecule inhibitors (Schiebel et al., 2013; Abrahams et al., 2016; Kumar et al., 2018; Cunningham et al., 2020; Inoyama et al., 2020) have provided atomic-level mechanistic insights into the KasA structure. In the crystals, KasA exhibits C2 symmetry and is composed of a five-layer αβαβα structure, a fingerprint of thiolases (**Figure 3**). Based on our understanding of KasA structure and function, and for the benefit of discussion, it can be divided into two regions – the core and cap domains. Within the core domain lies the catalytic triad residues (Cys171, His311, and His345) and the phoshopantetheine tunnel, which opens into the malonyl binding region. The proposed role of the catalytic triad in KasA acylation, decarboxylation and condensation is described in **Figure 4**. The acyl channel is a hydrophobic channel in the cap region formed by helices α2, α5, α9 and α5′. It can be accessed either through the malonyl binding site or from the opening of acyl channel in the cap region. It accommodates the growing acyl chain. The acyl chain binding site is traced by the phospholipid bound to KasA in PDB ID 4C6W (Schiebel et al., 2013). The cap domain is disordered in the apo structure but becomes ordered upon acylation of KasA Cys171 (Luckner et al., 2009; Kumar et al., 2018). The cap domain is proposed to be responsible for significant functional differences between KasA and KasB (Schiebel et al., 2013). How the core and cap domains function to condense the acyl donor and malonyl-AcpM substrates to form β-ketoacyl-AcpM products is outlined below.

In the apo state, the KasA active site residues are in a zwitterionic state, i.e., Cys171 is deprotonated and His311 is protonated. In step one, apo KasA binds to an acyl-AcpM, charging Cys171 with an acyl chain that is at least C_16-18_ and maximally C_36-40_ (Slayden and Barry, 2002). It is important to note that the acyl chain is covalently attached to AcpM *via* a phosphopantetheine group. Two models have been proposed to explain how the acyl chain accesses the KasA active site. In model one, it is hypothesized that both the acyl-AcpM and the malonyl-AcpM substrates are delivered through the phosphopantetheine tunnel mediated by the binding of acidic AcpM residues to basic residues on the KasA surface (namely Arg74, Arg 78, Arg79, Arg 135, Arg214 and Lys136) (Lee et al., 2011; Schiebel et al., 2013). In model two, it is proposed that KasA residues 115 – 147 in the flexible cap region of both monomers move in a scissor-like motion to allow direct access of the acyl chain into the hydrophobic cavity (Luckner et al., 2009). A structure of KasA in complex with acyl-AcpM may explain how the complex overcomes a variety of steric factors and the hydrophilic and hydrophobic nature of the acyl and phosphopantetheine channels. Structure-function studies do suggest that acylation of Cys171 likely induces a conformational change of gatekeeper residue Phe404 along with the additional gatekeeper residues Leu116 and Tyr126 (Schiebel et al., 2013). Acylated KasA adopts an open conformation, which facilitates the binding of malonyl-AcpM by widening the phosphopantethiene tunnel entrance and increasing the size of the malonyl binding site. Decarboxylation of malonyl-AcpM is then mediated by His311 and His345. It is important to note that different theories have been proposed to explain the protonation states of the catalytic triad residues during acylation and decarboxylation (Lee et al., 2011; Lee and Engels, 2014). Subsequent Claisen condensation occurs by a nucleophilic attack of the enolate on the thioester intermediate, yielding the product β-ketoacyl-AcpM (**Figure 4**).

## The thiolactomycin chemotype as a malonyl binding site inhibitor

**Figure 5:**
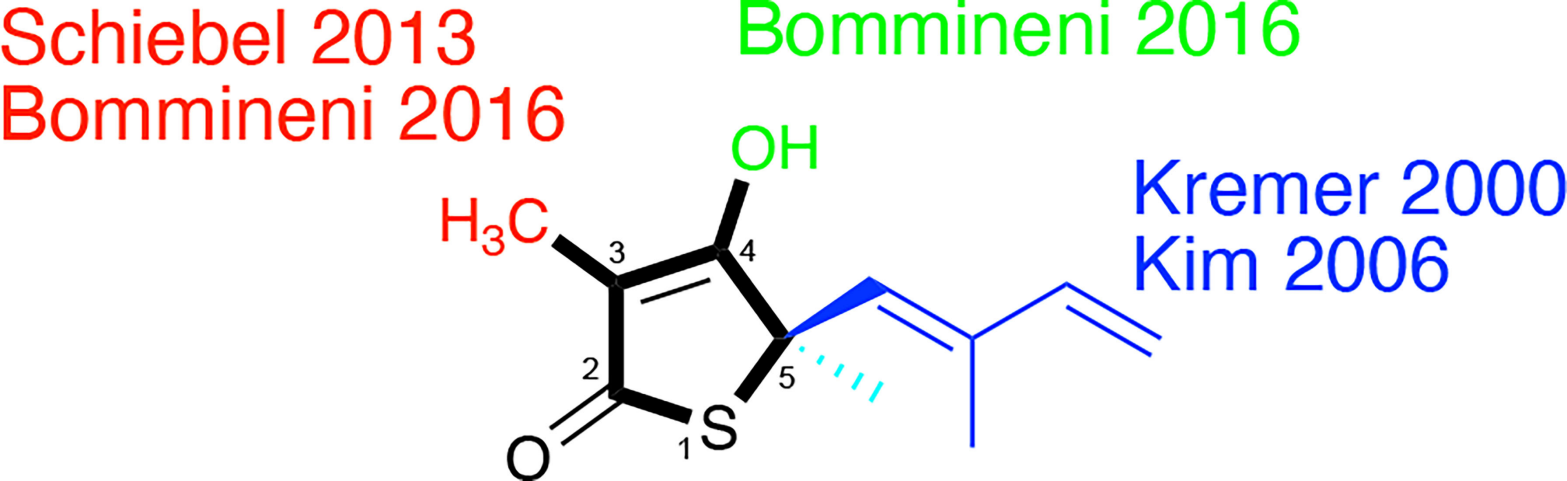
Thiolactomycin and its analogs. Noted are efforts, referenced within, regarding analogs at the specific positions of the thiophen-2(5H)-one ring system which are numbered.

Thiolactomycin (TLM; **Figure 5**) is a thiolactone natural product, isolated from *Nocardia* spp., that initially was noted for its modest *in vitro* growth inhibitory efficacy versus a range of Gram-positive and Gram-negative bacteria (Oishi et al., 1982). TLM has been shown to exhibit low cytotoxicity to mammalian cells. Analysis of TLM serum levels in orally dosed rats evidenced rapid absorption and clearance (Miyakawa et al., 1982). Accordingly, TLM and its analogs have exhibited only modest *in vivo* efficacy in mouse models of infection with *Staphylococcus aureus*, *Klebsiella pneumoniae*, and *Serratia marcescens* (Miyakawa et al., 1982; Bommineni et al., 2016).

Slayden et al. demonstrated TLM *in vitro* efficacy versus *M. tuberculosis* albeit with racemic, synthetic material with a MIC = 120 µM on solid media (Slayden et al., 1996); the active enantiomer features a 5*R*-stereocenter (Kim et al., 2006). This group also showed that TLM afforded bactericidal efficacy against *M. tuberculosis* in an infected murine bone marrow-derived macrophage model and inhibition of mycolic biosynthesis within *M. smegmatis*. Knowledge of its inhibition of *E. coli* β-ketoacyl synthases (Nishida et al., 1986; Magnuson et al., 1993) hinted at its engagement of KasA and KasB which was evidenced through overexpression studies in *M. bovis* BCG (Kremer et al., 2000). Studies with the purified *M. tuberculosis* proteins showed that TLM inhibited all three annotated *M. tuberculosis* β−ketoacyl synthases in various functional assays. Whereas the metrics of inhibition vary according to the assay, in general, TLM inhibition followed the order of KasA > KasB > FabH (Choi et al., 2000; Schaeffer et al., 2001; Kim et al., 2006; Machutta et al., 2010).

In 2000, Kremer et al. reported a subset of TLM analogs designed without the initial guidance from an X-ray crystal structure (Kremer et al., 2000). Their assay results demonstrated the importance of the TLM isoprenyl group in maintaining whole-cell activity against *M. tuberculosis* as judged by MIC but showed potential disconnects between MIC and the respective inhibitions of mycolic acid biosynthesis and FAS-II biosynthesis in *M. smegmatis*. While the authors constructed a KasA homology model from *E. coli* FabF (PBD ID: 1KAS) (Huang et al., 1998), their analysis was limited to the observation of a hydrophobic pocket that could be engaged by the isoprenyl. This observation would later be supported by a 2009 reported X-ray crystallographic study (Luckner et al., 2009) that illustrated the orientation of the isoprenoid moiety toward an extended pocket where two water molecules were present stabilizing the loop from Asp272 to Pro280. Furthermore, a thorough examination of a range of substitutions for the isoprenyl moiety was performed by Kim and colleagues (Kim et al., 2006). In an assay quantifying KasA catalytic activity, the authors found linear or branched alkyl, cycloalkyl, and aryl moieties failed to afford TLM analogs with inhibition within six-fold of TLM. In fact, all but two compounds demonstrated IC_50_ values in excess of 100 µM. Importantly, all analogs failed to exhibit significant *M. tuberculosis* growth inhibitory efficacy versus the H37Rv strain.

**Figure 6:**
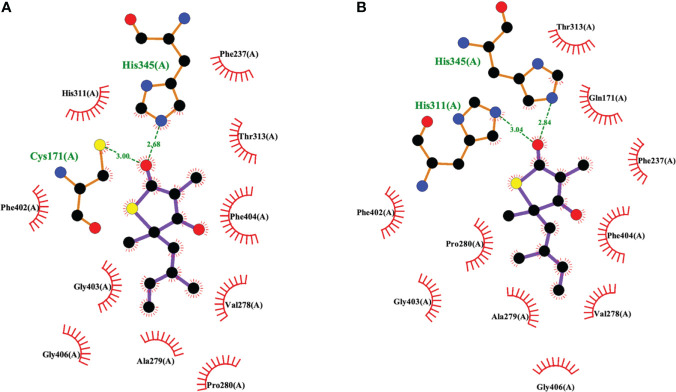
Interaction profiles for TLM bound to **(A)** wild type KasA (PDB ID 2WGE) and **(B)** KasA-C171Q (PDB ID 2WGG). Hydrogen bonds are shown as green dashed lines and distances are measured in Angstroms.

The 2009 structural biology report (Luckner et al., 2009) was critical to elucidating the details of how TLM binds to KasA (**Figures 3**, **6**). More specifically, it illustrated how TLM occupies the malonyl binding site. TLM interactions with the active site residues and the binding mode of TLM itself differ when KasA is in the closed (apo) or open (acylated) conformations (PDB IDs 2WGE and 2WGG, respectively). The isoprenoid chain and the double bond in the thiophen-2(5H)-one of TLM are critical for KasA inhibitory activity. In apo KasA, the TLM carbonyl oxygen formed two hydrogen bonds with the sulfhydryl group of Cys171 and sidechain N-H of His345, whereas in the C171Q mutant (that mimics acylated KasA) two hydrogen bonds were formed with the sidechain N-H moieties of His311 and His345. Furthermore, in the apo state, the KasA phenyl ring of gatekeeper residue Phe404 formed a face-to-face interaction with the thiolactone ring. With the KasA C171Q mutant, however, a conformational change shifted the Phe404-TLM interaction to edge-to-face. The orientation of the TLM isoprenoid tail in the lipophilic pocket also varied between the apo KasA and KasA-C171Q structures. Movement of Phe404 from the closed to open conformation led to a shift in residues Leu116, Val142, and Met146, and helix α9. This conformational change increased the size of the malonyl binding site and the phosphopantetheine tunnel. This explains the slow (Machutta et al., 2010) and preferential binding of TLM to acylated KasA as compared to apo KasA.

**Figure 7:**
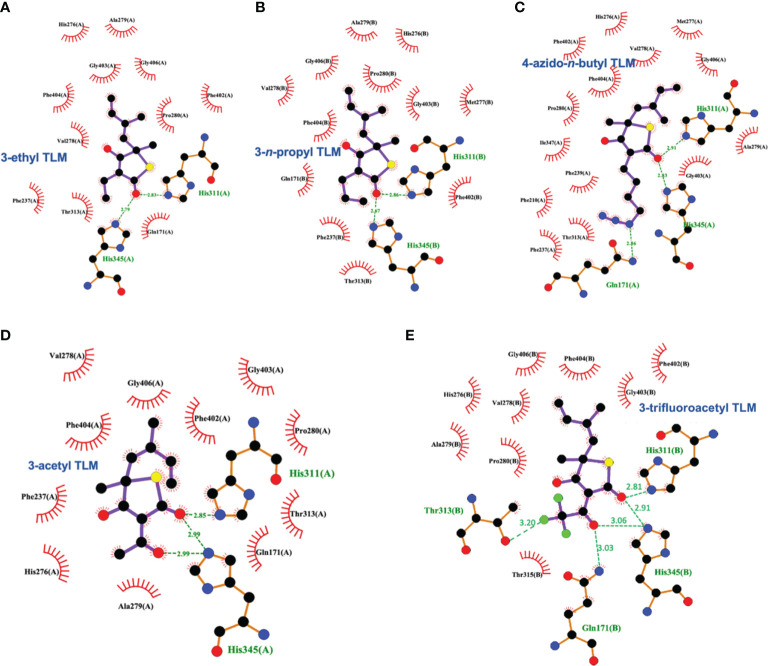
Interaction profiles for KasA C171Q bound to the following TLM analogs: **(A)** 3-ethyl (PDB ID 4C6Z), **(B)** 3-*n*-propyl (PDB ID 4C70), **(C)** 4-azido-*n*-butyl (PDB ID 4C71), **(D)** 3-acetyl (PDB ID 4C72), and **(E)** 3-trifluoroacetyl (PDB ID 4C73). Hydrogen bonds are shown as green dashed lines and distances are measured in Angstroms.

Schiebel and colleagues leveraged this structural information in the pursuit of TLM analogs (Schiebel et al., 2013). Attempting to mimic the alkyl portion of the acyl chain of bound acyl-AcpM, linear hydrophobic substituents (e.g, ethyl, *n*-propyl, and 4-azido-*n-*butyl) were introduced at the 3-position of the thiolactone ring (**Figure 5**). The TLM alkyl-AcpM mimics displayed a 4 – 18 fold reduction in KasA binding affinity for the C171Q mutant in comparison to TLM. However, they did maintain the TLM slow onset of binding phenotype. X-ray crystal structures demonstrated how the introduced alkyl chain oriented towards the aromatic cavity formed by Phe210, Phe237, Phe239, and His345 (PDB IDs 4C70, 4C6Z, 4C71; **Figure 7**). Interestingly, upon comparison to the whole-cell efficacy of TLM versus the *M. tuberculosis* H37Rv strain, the 3-ethyl analog was approximately equipotent while the 3-*n*-propyl derivative was about eightfold less active (Bommineni et al., 2016). Kapilashrami continued this effort and their *para*-substituted phenethyl and phenylbutyl analogs generally offered enhanced binding affinity for wild type KasA and an improved initial equilibrium binding constant for the C171Q mutant as compared to TLM, but failed to exhibit slow binding kinetics (Kapilashrami et al., 2013). Slow onset of binding has been demonstrated to be a key aspect of long residence time inhibitors and to their *in vivo* efficacy (Lu and Tonge, 2008; Lu and Tonge, 2010). These results serve as a reminder of the intricacies associated with inhibitor binding to KasA and the value of detailed kinetic measurements. Furthermore, an X-ray crystal structure of KasA bound to one of these 3-alkylphenyl analogs would help explain these observations.

TLM 3-substituted acyl analogs (e.g., acetyl and trifluoroacetyl) utilized the acyl moiety to mimic the diketo group of malonyl-AcpM. These derivatives offered 2 – 4 fold increased affinity for the C171Q mutant as compared to TLM while still maintaining slow onset kinetics. X-ray crystallographic studies again proved insightful (**Figure 7**). The structure of either 3-acyl analog is supportive of the increased binding affinity being due to the formation of a new hydrogen-bonding interaction between the acyl carbonyl and His345. A potentially favorable interaction between the trifluoromethyl group in the 3-trifluoroacetyl analog and the sidechain hydroxyl of Thr313 may also be present. In comparison to the whole-cell efficacy of TLM versus the *M. tuberculosis* H37Rv strain, the 3-acetyl and 3-trifluoroacetyl analogs were 4- and 32-fold less potent, respectively (Bommineni et al., 2016). Kapilashrami reported additional acyl analogs with a longer alkyl chain (i.e., *n*-Pr, C_15_H_31_, (CH_2_)_5_(4-PhC_6_H_4_)). These were not slow onset binders and they failed to offer improvements in binding affinity to wild type KasA or initial equilibrium binding constant for the C171Q mutant. These results further support the value of an X-ray crystal structure of KasA bound to malonyl-AcpM, or a non-reactive mimic.

Removal of the TLM 3-methyl group was also pursued and led to a decrease in whole-cell potency (MIC > 100 µM) while providing gains in the binding constants for the wild type and C171Q forms of KasA, respectively (Bommineni et al., 2016). The disconnect between enzyme binding and whole-cell efficacy could potentially be further explored by examining the effect of methyl group removal on compound accumulation and/or metabolism within *M. tuberculosis* (Wang et al., 2019; Wang et al., 2020). Furthermore, alkylation of the 4-OH of this desmethyl TLM abrogated not only growth inhibition of *M. tuberculosis* but also binding to wild type KasA or KasA-C171Q. An observed decrease in potency *via* removal of the hydroxy group may be consistent with the loss of a potential water-mediated hydrogen bond with the carbonyl oxygen of Val278. It is also possible that the newly introduced alkyl substituent makes unfavorable interactions with nearby KasA residues. While analysis of the relevant crystal structures does not reveal obvious unfavorable interactions, it is, however, possible that unfavorable interactions could occur when KasA adopts different conformations, e.g., when complexed with AcpM.

## The indazole sulfonamide chemotype as an acyl channel inhibitor

**Figure 8:**
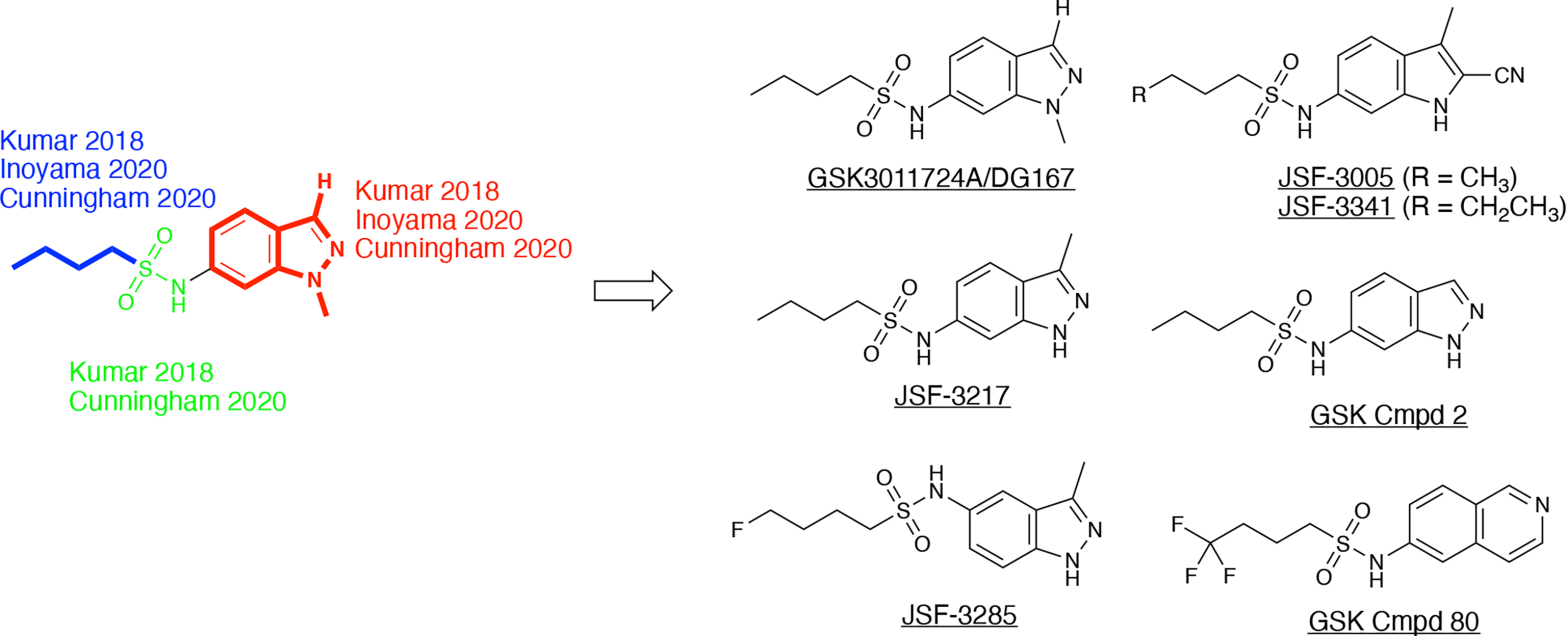
DG167 and its analogs. The derivatives, as referenced herein, are noted with regard to alterations in the heterocyclic core and sulfonamide moiety.

GSK3011724A, an indazole sulfonamide (**Figure 8**), was first identified as an antitubercular by GlaxoSmithKline (GSK) through a high-throughout whole-cell phenotypic screening campaign (Ballell et al., 2013; Rebollo-Lopez et al., 2015). This compound and other screening actives were made available to the research community to explore their mechanism of action and optimization. GSK and our laboratory (renaming the molecule as DG167; a convention that will be used throughout this review) each reported on their respective initial studies which characterized the property profile and mechanism of action of this indazole sulfonamide (Abrahams et al., 2016; Kumar et al., 2018). In our case, we were drawn to DG167 because of its signature as an inhibitor of cell wall biosynthesis through its induction of *iniBAC (* Wilson et al., 2013) that lacked cross-resistance with current front- and second-line tuberculosis drugs. While the two reports differ in terms of their exact values for different property metrics, in general, DG167 exhibited promising *in vitro* (i.e., sub-micromolar) potency versus the *M. tuberculosis* H37Rv strain, a lack of cytotoxicity to model mammalian cell lines, and good aqueous solubility. Both groups determined through the generation of spontaneous resistant mutants that DG167 targets KasA with acceptable frequencies of resistance for an antitubercular drug discovery molecule. The protein target was further confirmed *via* additional techniques, such as FAS-II thin layer chromatography (TLC) to evidence mycolic acid biosynthesis inhibition, binding constant quantification with respect to purified KasA, functional inhibition of purified KasA, and target pull-down with a bead-bound analog of DG167.

**Figure 9:**
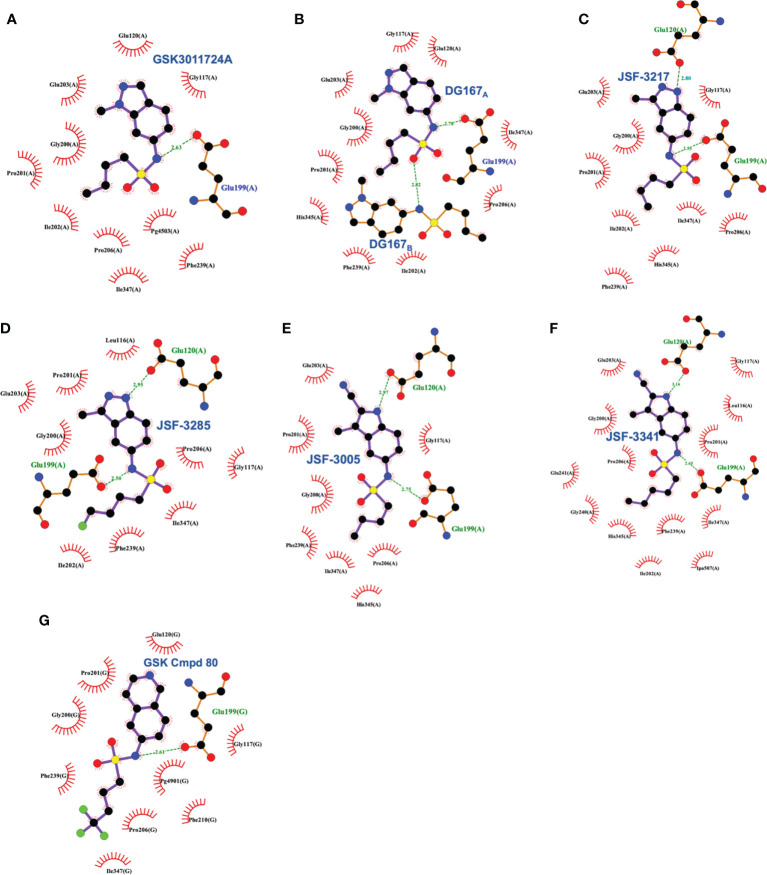
Interaction profiles for wild type KasA bound to the following sulfonamides: **(A)** GSK3011724A (PDB ID 5LD8), **(B)** DG167 (PDB ID 5W2P), **(C)** JSF-3217 (PDB ID 5W2S), **(D)** JSF-3285 (PDB ID 6P9L), **(E)** JSF-3005 (PDB ID 6P9K), **(F)** JSF-3341 (PDB ID 6P9M), and **(G)** GSK Cmpd 80 (PDB ID 6Y2J). Hydrogen bonds are shown as green dashed lines and distances are measured in Angstroms.

The X-ray crystal structure of this compound has been elucidated bound to KasA (Abrahams et al., 2016; Kumar et al., 2018). The GSK report established the initial crystal structure (PDB ID 5LD8) and our work had significant contributions in confirming and correcting the initial findings (PDB 5W2P). The GSK structure depicted the acyl channel of each KasA subunit to be occupied by one molecule of DG167 and one molecule of polyethylene glycol (PEG). The purification and crystallization conditions, however, excluded PEG. It is, therefore, unclear to us why PEG was modeled in this structure and continued to be modeled in the more recently determined structures of KasA (Cunningham et al., 2020). In contrast, the electron density maps of our published structure of KasA-DG167 enabled us to unambiguously build two molecules of DG167 (DG167_A_ and DG167_B_) in the acyl binding site (**Figures 3**, **9**). DG167_B_ was in the position modeled as PEG in the GSK publication. KasA-DG167 binding was stabilized by both hydrophobic and hydrogen bond interactions. For instance, the DG167_A_ sulfonamide N-H formed a hydrogen bond with the Glu199 sidechain. An intermolecular hydrogen bond was observed between the DG167_A_ sulfonamide oxygen and the DG167_B_ sulfonamide N-H. Both indazole units of DG167_A_ and DG167_B_ contributed to hydrophobic interactions throughout the acyl channel. We observed the aliphatic moiety of DG167_A_ mirrored the binding of the phospholipid acyl tail reported in previously determined structures of KasA (e.g., PDB ID 4C6W) (Schiebel et al., 2013). More specifically, this *n-*butyl moiety of the 6-sulfonamide was bound in a narrow hydrophobic channel lined by Gly200, Ile202, Pro206, Phe239, His345, and Ile347. Furthermore, the 1-methyl group fit into a shallow pocket defined by Pro201, Glu203, and Pro206, and engaged these amino acids through hydrophobic interactions. The DG167_B_ indazole nitrogen formed water-mediated hydrogen bonds with Gly115, Asn148, and Ala170. Additionally, the DG167_B_ indazole mediated hydrophobic interactions across the KasA/KasA′ dimer interface. It is important to note that in the KasA-DG167 structure, gatekeeper residue Phe404 was in the closed conformation where acyl chain access to the acyl channel was restricted. This was, to our knowledge, the first time a KasA ligand (such as an inhibitor) was demonstrated to bind KasA in the closed (nonacylated) conformation. It is remarkable that DG167 can access the acyl channel while Phe404 is in the closed conformation, defeating the elaborate mechanisms KasA has evolved to prevent the entry of cellular free fatty acids into the acyl channel.

With the target of DG167 firmly established as KasA, the question as to its *in vivo* profile arose. Studies in both laboratories found the compound to exhibit a modest pharmacokinetic (PK) profile in mice with some evidence of toxicity at doses ≥300 mg/kg once-daily oral (qd po). We proposed that the oral exposure was limited by demethylation of the 1-methyl moiety (half-life t_1/2_ = 10.1 min) to afford the whole-cell inactive 1H-indazole, as observed through incubation of DG167 in the presence of mouse liver microsomes (MLM). Consistent with its modest PK profile, we did not observe DG167 (100 mg/kg qd po) to exhibit the ability to reduce *M. tuberculosis* infection in a sub-acute model of infection when quantifying bacterial burden in the lungs of female BALB/c mice dosed for 2 weeks as compared to the lung bacterial burden at the outset of compound treatment. The GSK report first profiled DG167 in a “fast” and less conservative model (Rullas et al., 2010) with 8 d of drug treatment commencing 1 d post infection of C57BL/6 mice. Quantification of the reduction of lung bacterial burden utilized the level in mouse lung for the no-drug control arm at the end of treatment as the comparison. GSK reported cidal activity in this infection model as well as in a more typical chronic model of infection albeit with what we would term a less conversative calculation of bacterial load reduction.

These *in vivo* results, in our minds, formed the basis of an optimization problem where we hypothesized that DG167 analogs with enhanced metabolic stability and mouse PK profile would demonstrate significant bactericidal efficacy *in vivo*. In our structure-based optimization reported in 2020, the central design hypothesis was to transpose the indazole 1-nitrogen of DG167 to afford 3-methyl-5-sulfonamide indazoles or indoles (Inoyama et al., 2020). It was hypothesized that this would maintain the hydrophobic interactions of the DG167 1-methyl moiety that our 2018 publication (Kumar et al., 2018) had demonstrated could not be replaced without losses in whole-cell potency and surprisingly also in MLM stability. We entertained a small number of potential alterations to the sulfonamide substituent, having learned from our earlier work (Kumar et al., 2018) that *n-*alkyl groups larger than pentyl and smaller than butyl afforded poorer MIC values. Furthermore, consistent with the relative narrowness of the hydrophobic channel recognizing the sulfonamide alkyl group, we were cognizant that substitutions on all but the terminal carbon of the *n-*butyl, or its replacement with carbocyclic or aromatic/heteroaromatic groups, were not tolerated. It should be noted that we had preliminarily reported on one transposed indazole (compound 5g or JSF-3217; **Figure 8**) in our 2018 publication that exhibited greater whole-cell activity (MIC = 0.2 µM) than DG167 but with only marginally better MLM stability (t_1/2_ = 11.5 min). Critically, an X-ray structure of JSF-3217 bound to KasA (PDB ID 5W2S) demonstrated the protein-inhibitor interactions that were in our initial designs (**Figure 9**). Analysis of the X-ray crystal structure led us to postulate that a second molecule of JSF-3217 cannot bind in the DG167_B_ site because it would force the indazole N(1)-H in close proximity to the hydrophobic surface associated with the KasA dimer interface. In addition to the sulfonamide N-H hydrogen bond with Glu199 similar to the one observed in the DG167 structure, the N-H group of the transposed indazole formed a new hydrogen bond with Glu120. Only one molecule of JSF-3217 bound to a KasA monomer and did not contact the other protomer in the KasA dimer. The KasA–JSF-3217 structure also showed that single molecule acyl channel occupancy was sufficient to stabilize the KasA flap (Luckner et al., 2009) (residues 115 – 147). Further optimization efforts with the transposed indazoles culminated in JSF-3285, which replaced the *n*-butyl of JSF-3217 with a 4-fluorobutyl. The main improvements realized with JSF-3285 as compared to DG167 were its improved mouse PK profile (AUC_0-5h_ = 59323 vs. 1965 h*ng/mL), MLM stability (t_1/2_ = 28.4 vs. 10.1 min), and kinetic aqueous solubility (S = 483 vs. 324 µM) while slightly enhancing *in vitro* potency (MIC = 0.20 vs. 0.39 µM). Critically, the *in vitro* efficacy of JSF-3285 versus the H37Rv laboratory strain was maintained versus a set of 48 drug-sensitive and drug-resistant clinical strains. This observation furthers our confidence that a KasA-targeting therapeutic will be of significant utility versus both drug-sensitive and drug-resistant infections. We determined the X-ray crystal structure of KasA complexed with JSF-3285 (PDB ID: 6P9L) (**Figure 9**). Overall, the JSF-3285 and JSF-3217 binding modes were similar. The JSF-3285 sulfonamide alkyl moiety, however, reached further into the hydrophobic channel, making different hydrophobic contacts than the JSF-3217 alkyl sulfonamide. Overall, we were gratified to have structural data to support our structure-based design hypothesis; removal of the metabolic instability of DG167 afforded an advanced compound for further study.

In addition to the transposed indazole optimization campaign, our analysis of the X-ray structure of the KasA-DG167 structure supported removal of the N(2) from the transposed indazole design to afford 3-methyl-5-sulfonamide indoles where we could explore additional interactions with KasA through modification of the 2-substituent. Key molecules in this campaign were JSF-3005 and JSF-3341 (**Figure 8**) which both featured a 2-cyano group and the sulfonamide moiety as *n-*butyl or *n-*pentyl, respectively. Their *in vitro* growth inhibitory potencies versus the *Mtb* H37Rv strain were 0.78 and 0.20 µM, respectively. Each indole was crystallized bound to the KasA (**Figure 9**). Both JSF-3005 and JSF-3341 utilized similar hydrogen bonding interactions between the indole N-H and KasA Glu120, as well as the sulfonamide N-H and KasA Glu199. However, a few differences were observed like additional electron density corresponding to an unknown molecule identified in the acyl channel of the KasA–JSF-3005 complex. The alkyl chain in JSF-3341 is longer by one carbon than in JSF-3005, extending 0.9 Å deeper into the hydrophobic channel. The sulfonamide group in JSF-3341 was slightly shifted by 0.5 Å when compared to JSF-3005. The additional methyl group on the sulfonamide of JSF-3341 also mediated additional hydrophobic contacts not observed in the KasA–JSF-3005 complex structure.

JSF-3005, JSF-3341, and JSF-3285 were studied thoroughly in an effort to proceed to *in vivo* efficacy assessment. Summarily, all three compounds were rigorously confirmed to primarily target KasA through the following methods in addition to the previously described X-ray crystallography with their KasA complex: quantification of KasA binding *via* microscale thermophoresis, fatty acid TLC, and drug-resistant mutant generation and sequencing. The three molecules were assessed in mouse models of sub-acute and chronic *M. tuberculosis* infection. In the sub-acute model with four weeks of compound treatment, JSF-3005 dosing (100 mg/kg qd po) led to increases in the lung bacterial burden and JSF-3341 dosing (200 mg qd po) afforded bacteriostatic activity. JSF-3285 was dosed at 100 mg/kg and 200 mg/kg qd po, in accordance with dose proportionality and tolerability studies, in the sub-acute infection model, and we were delighted to observe an ca. 2 log_10_ reduction in bacterial burden as quantified by the lung bacterial burden post 4 weeks of treatment as compared to the bacterial burden at the beginning of compound treatment. The corresponding chronic infection assessment in BALB/c mice demonstrated JSF-3285 at 100 or 200 mg/kg to reduce the bacterial burden in the lungs >2 log_10_ colony-forming units (CFUs) after 4 weeks as compared to the bacterial burden at the start of the treatment. The addition of JSF-3285 (200 mg/kg) to INH or RIF (either at 10 mg/kg) improved efficacy of the front-line drug by about 1 log_10_ CFUs. Furthermore, the bactericidal efficacy of JSF-3285 at doses ranging from 200 – 20 mg/kg qd po was examined. At 20 mg/kg qd po, INH and RIF each afforded just more than a 2 log_10_ reduction in CFUs. JSF-3285 exhibited an approximately 1.5 – 2.0 log_10_ reduction in CFUs at doses of 20 – 200 mg/kg. These experiments provided rigorous validation for JSF-3285 as a preclinical tuberculosis drug candidate and critically established *in vivo* pharmacological validation of KasA with conservative quantification of cidal bacterial efficacy.

Building on their earlier work (Abrahams et al., 2016), a GSK team reported (Cunningham et al., 2020) on the results of their evolution of DG167 in a manuscript submitted four months after the submission of our report; both papers were published in March 2020. They verified the whole-cell potential of the transposed indazole chemotype found by us (Kumar et al., 2018; Inoyama et al., 2020), but mainly focused on their finding of the Ames mutagenicity of the amine metabolite of their initial hit or select transposed indazoles (e.g., GSK Cmpd 2 in **Figure 8**). Our efforts to address this concern will be published separately. While the amount of the amine metabolite found in the urine of a single Sprague Dawley rat dosed at 300 mg/kg po in each case was not quantified, the *in vivo* observation of an amine with an Ames positive signal was a significant concern in the absence of further studies.

The GSK team proposed several strategies to prevent the formation of a mutagenic amine, primarily by substituting for the sulfonamide with other moieties that would not suffer hydrolysis. Additionally, the steric and/or electronic environment was modified around the 6-membered ring of the core heterocycle or the 5-membered ring of the indazole was replaced with other ring systems; the goal was to eliminate the mutagenicity of the amine formed. These alterations failed to find afford a coalescence of antitubercular whole-cell efficacy with a lack of Ames mutagenicity associated with the related amine metabolite.

A significant factor in this outcome was the necessity of maintaining the sulfonamide, which, as described earlier, serves as a recognition element providing an appropriate straight-chain alkyl hydrophobe along with the hydrogen bond between its N-H to Glu199. Changes of the central heterocycle from indazole to indole, 2,3-dihydro-1*H*-indene, and isoquinoline (e.g., GSK Cmpd 80 in **Figure 8**) were achieved, but they do not appear to have offered sufficient advantages worthy of further exploration. The indole substitution was not surprising given that it involves the removal of the indazole nitrogen from DG167 to afford a 5-substituted indole. The corresponding N-(2,3-dihydro-1H-inden-5-yl)butane-1-sulfonamide exhibited modest efficacy (MIC = 16.3 µM) while lacking the N-H to proposedly hydrogen bond with Glu120. Finally, the GSK Cmpd 80 isoquinoline sulfonamide demonstrated modest potency (MIC = 15 µM) and was structurally characterized bound to KasA (PDB ID 6Y2J; **Figure 9**). While its *n-*butyl sulfonamide exhibited similar interactions as DG167, its basic nitrogen was shown to engage a water molecule through hydrogen-bonding; Glu120 did not interact with the inhibitor. These results led the authors to speculate on the need for the planarity of the heterocycle given the proximity of Gly200 and Pro201. However, it is not obvious that this would contribute significantly to the binding energy. We suspect that the loss of hydrogen-bonding to Glu120 is primarily responsible for the reduction in compound activity.

## Summary

This review focuses on the promise of KasA as a tuberculosis drug target and, in particular, on the importance of structure-based design approaches to deliver KasA inhibitors of translational significance. Thus, we did not discuss in-depth earlier stage molecules, such as cerulenin (Schaeffer et al., 2001) and platensimycin (Brown et al., 2009), that have not been supported by published X-ray crystal structures in complex with KasA. We also have not described computational approaches (Puhl et al., 2020) involving docking and/or machine learning methods to design KasA binders. Most likely, X-ray crystal structures of these compounds bound to KasA will be necessary to enable their design optimization. Our analysis of the literature supports KasA as a highly valuable target for future study. A panoply of approaches has been utilized to vet KasA with regard to its essentiality and vulnerability. Two binding sites (i.e., the malonyl binding and acyl binding sites) have been targeted with small molecules and the relevant interactions have been discerned through X-ray crystallography. KasA acyl binding channel targeting with drug-like small molecules has been shown to be critical to significantly reduce the *M. tuberculosis* infection in mice, while efforts with malonyl binding site inhibitors have fallen short thus far of *in vivo* pharmacologic validation. While the current *in vivo* active molecules have limitations, we expect that ongoing studies with JSF-3285 and newer series from our laboratories, GSK, and others will find the requisite balancing of efficacy and toxicity profiles. We anticipate that structure-based design efforts, as summarized herein, will play a prominent role in these endeavors. Ultimately, we are optimistic that the sum total of efforts with *M. tuberculosis* KasA inhibitors will afford one or more molecules for clinical studies that will positively impact drug regimens for both drug-sensitive and drug-resistant infections.

## Author contributions

All authors listed have made a substantial, direct, and intellectual contribution to the work, and approved it for publication.

## Funding

This work was supported at Rutgers University – New Jersey Medical School by the US National Institutes of Health grants U19AI142731 (MN and JF).

## Acknowledgments

We want to thank Dr. Clifton E. Barry III (NIH/NIAID) and Dr. Alexander L. Perryman (Repare Therapeutics) for helpful discussions.

## Conflict of interest

MN and JF are listed as inventors on patent filings pertinent to the indole and indazole compounds mentioned in this manuscript as employees of Rutgers University.

The remaining authors declare that the research was conducted in the absence of any commercial or financial relationships that could be construed as a potential conflict of interest.

## Publisher’s note

All claims expressed in this article are solely those of the authors and do not necessarily represent those of their affiliated organizations, or those of the publisher, the editors and the reviewers. Any product that may be evaluated in this article, or claim that may be made by its manufacturer, is not guaranteed or endorsed by the publisher.
